# Prognostic value of adiponectin level in patients with coronary artery disease: a systematic review and meta-analysis

**DOI:** 10.1186/s12944-019-1168-3

**Published:** 2019-12-23

**Authors:** Lili Yang, Bin Li, Yuan Zhao, Zhengyi Zhang

**Affiliations:** Department of General Medicine, Lanzhou University Second Hospital, No. 82 Cuiyingmen, Lanzhou, 730030 Gansu Province China

**Keywords:** Coronary artery disease, Cardiovascular events, Cardiovascular mortality, All-cause mortality, Meta-analysis

## Abstract

**Background:**

Conflicting results on the prognostic value of blood adiponectin level in patients with coronary artery disease (CAD) have been reported. This meta-analysis aimed to investigate the prognostic value of elevated adiponectin level in CAD patients.

**Methods:**

A comprehensive literature search was conducted in PubMed and Embase databases up to May 10, 2019. Studies evaluating the association between adiponectin level and major adverse cardiovascular events (death, stroke, acute coronary syndrome or coronary revascularisation), cardiovascular mortality, and all-cause mortality in CAD patients were included. Pooled multivariable adjusted risk ratios (RR) and 95% confidence intervals (CI) was calculated for the highest vs the lowest category of adiponectin level.

**Results:**

Twelve studies including 10,974 CAD patients were included. Elevated adiponectin level was independently associated with higher risk of cardiovascular (RR 1.93; 95% CI 1.55–2.42; *p* < 0.001) and all-cause mortality (RR 1.96; 95% CI 1.64–2.34; *p* < 0.001) in CAD patients. However, CAD patients with higher adiponectin level did not significantly increase major cardiovascular events risk (RR 1.12; 95% CI 0.86–1.45; *p* = 0.407) after adjustment for potential confounders.

**Conclusions:**

This meta-analysis indicates that elevated adiponectin level is an independent predictor of cardiovascular and all-cause mortality in CAD patients. Measurement of blood adiponectin level has potential to identify CAD patients who have high risk of death.

## Backgrounds

Coronary artery disease (CAD) is the most common type of heart disease. In spite of advance in medical science, CAD cannot be completely prevented and avoided. Patients with CAD are under threat of subsequent cardiovascular events. CAD remains the globally leading cause of death among men and women [1]. However, traditional biomarkers are insufficient to predict the secondary cardiovascular events in these patients. Therefore, identification of novel predictive biomarkers is clinically urgent need for more intensive secondary prevention of cardiovascular events [2].

Biomarkers are frequently used for diagnosis and risk stratification of CAD. Adiponectin is an adipocyte-specific cytokine secreted predominantly from adipocytes [3]. Hypoadiponectinemia and hyperadiponectinemia are associated with various diseases [4]. Epidemiologic studies of healthy individuals and patients with established cardiovascular disease have suggested an association between elevated circulating adiponectin level and increased risk of cardiovascular and all-cause mortality [5, 6]. In the presence of existing CAD, circulating adiponectin level could also serve as a potential prognostic biomarker in these patients [7–11]. However, there are controversial findings on the prognostic value of elevated adiponectin level for predicting cardiovascular events and mortality in patients with CAD [12–14]. Nevertheless, the magnitude of the prognostic value varied among these studies.

No previous systematic review or meta-analysis has specially focused on the prognostic value of elevated adiponectin level in CAD patients. Given these heterogeneous findings, we performed this meta-analysis to investigate the prognostic role of elevated adiponectin level for predicting MACE and survival outcomes in CAD patients.

## Methods

### Literature search

This meta-analyses followed the Preferred Reporting Items for Systematic Reviews and Meta-analyses (PRISMA) [15]. Two authors independently searched the articles indexed in PubMed and Embase databases from their inceptions to May 10, 2019. Search keywords used for literature search included: “adiponectin” AND “coronary artery disease” OR “coronary heart disease” OR “myocardial infarction” OR “acute coronary syndromes” OR “angina” AND “death” OR “mortality” OR “cardiovascular events”. The detaied search strategy is showed in Additional file 1. Additionally, reference lists of included studies and related reviews were also manually reviewed to identify additional studies.

### Study selection

Studies satisfied the all the following criteria were included: 1) original longitudinal studies enrolling CAD patients, 2) baseline blood adiponectin level as exposure, and, 3) reported multivariable adjusted risk ratios (RR) or hazard ratios (HR) or odds ratio (OR) with their corresponding 95% confidence intervals (CI) of all-cause or cardiovascular mortality and/or major adverse cardiovascular events (MACE) for the categorical adiponectin level during the follow-up. Exclusion criteria included the following: 1) population were not restricted in CAD patients or coexisting with CAD and other specific diseases, 2) reported risk estimate by continuous adiponectin level, 3) provided unadjusted risk estimate, and, 4) meeting abstracts, commentaries, reviews or duplicate publications.

### Data extraction and quality assessment

Two authors independently extracted the data and evaluated the study quality. *Disagreements* among two authors in data extraction and quality assessment were resolved through discussion. Data extracted from the eligible studies included first author’s surname, year of publication, country/region, study design, type of CAD, number of participants, percentage of men, mean age or age range at enrollment, cutoff value of adiponectin, mean or median follow-up, type of outcome, number of events, fully adjusted risk estimate, and adjustment of variables in the multivariate analysis. The methodological quality of all eligible studies was evaluated using the nine-star Newcastle-Ottawa Scale (NOS) for cohort studies [16], which bases its assessment on selection of the study groups, comparability of study groups, and ascertainment of the outcome. Studies awarding 7 stars or over were deemed as high quality.

### Statistical analysis

All statistical analyses were performed using STATA 12.0 (STATA Corp LP, College Station, TX, USA). The multivariable adjusted risk estimate was pooled for the highest vs the lowest category of adiponectin level. For studies reporting risk estimate by the lowest vs the highest adiponectin level, we recalculated it for the highest vs the lowest category. Heterogeneity among studies was quantitatively assessed using the Cochrane Q statistic (*p* < 0.10, statistically significant heterogeneity) and the I^2^ statistic (≥50%, statistically significant heterogeneity). A random effect model was applied in the case of significant heterogeneity. Otherwise, we chose a fixed-effect model. Begg’s test [17] and Egger’s test [18] were used to examine publication bias, with a *p*-value < 0.10 suggesting statistical significance. Moreover, we scheduled a subgroup analysis according to sample sizes, type of CAD, length of follow-up, adjustment of left ventricular ejection fraction and NOS score. Sensitivity analysis was conducted by omitting one study at each turn to check the reliability of the pooling risk estimates.

## Results

### Search results and study characteristics

**Fig. 1:**
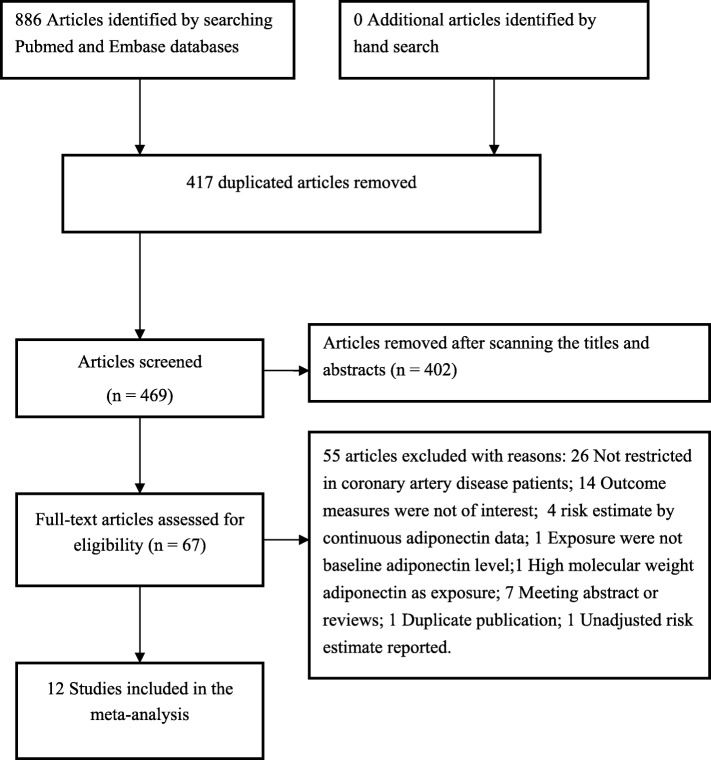
Flow chart of the study selection process

The study selection process is summarized in Fig. 1. Our initial literature search yielded 886 potentially relevant citations. Among the citations, 417 duplicates were excluded. After reviewing the titles and abstracts, 402 articles were excluded. The remaining 69 full-text articles were retrieved for detailed assessment. After removing all studies that did not satisfy the inclusion criteria, twelve studies [7–14, 19–22] were finally included in the current meta-analysis.

Table 1 summarizes the main characteristics of the included studies. All the included studies were prospective designs and published from 2006 to 2016. The sample size ranged from 77 to 3931, with a total of 10,974 CAD patients. The length of mean or median follow-up ranged from 12 months to 8.1 years. Overall, there were 1818 MACE, 842 all-cause and 285 cardiovascular death in the selected studies. Nine studies [7, 8, 11–14, 19–21] had a score of 7 stars or over (Table 2), indicating a relatively high quality.

**Table 1:** Main characteristic of the included studies

Author/year	Region	Study design	Patients (% male)	Mean age (years)	Outcome definition	Adiponectin cutoff	Follow-up (years)	Outcome measures HR or RR(95% CI)	Adjustment for variables
Pilz 2006 [7]	Germany	P	CAD; 2473 (75)	64 ± 10	–	Quartile 4 vs. 1; ≥14.77 vs. < 6.96 μg/ml	5.45	Total death:76; 2.04 (1.53–2.71); CV death:95 2.14 (1.52–3.02)	Age, gender, BMI, metabolic syndrome/type 2 DM, hypertension, smoking, LDL-C, HDL-C, TG, CRP, fibrinogen, eGFR, homocysteine, and NT-pro-BNP
Shioji 2007 [8]	Japan	P	Angina pectoris or acute MI; 184 (69.0)	66.2 ± 9.5	Death, re-infarction, revascularization, HF hospitalization, and CI	> 4.5 vs. ≤ 4.5 μg/ml	2.3	MACE:78; 0.35 (0.14–0.90) ^a^	Age, gender, BMI, fasting glucose, hemoglobin A1c, final reference diameter, DM, and medications
Piestrzeniewicz 2008 [9]	Poland	P	STEMI;77 (100)	54.3 ± 6.8	CV death, nonfatal MI, angina/HF hospitalization	≥4.23 vs. < 4.23 μg/ml	1.0	MACE:9; 5.08 (1.11–23.2)	DM, multivessel disease, LVEF CRP, history of CV events, lipids, creatinine, eGFR, hypertension, LVEF, and Duke Prognostic Index score
Maiolino 2008 [12]	Italy	P	CAD; 712 (72.5)	6.5 ± 10.1	CV death, nonfatal MI, ACS, stroke, and vascular surgery	> 6.38 vs. ≤ 6.38 μg/ml	3.8	CV death:45 1.37 (0.86–2.17) MACE:119; 1.00 (0.77–1.31)	Age, gender, LVEF, HD-C, LDL-C, BMI, creatinine, homocysteine, smoking, and TG
Huang 2010 [10]	Taiwan	P	AMI; 102 (85)	62 ± 11	CV death, nonfatal MI, PCI/CABG, IS, rehospitalization	≥6.46 vs. < 6.46 μg/ml	3.6	MACE:30; 1.22 (1.03–1.45)	Age, DM, hypertension, smoking, HDL-C, LDL-C, BMI and medication
Wilson 2011 [13]	Multi-coumtries	P	ACS;3931 (78.4)	48–70	Death, MI, stroke, unstable angina, CHF, revascularization	> 4.477 vs. ≤ 4.477 μg/ml	2.0	Total death:118; 1.57 (0.95–2.58); MACE:951; 1.20 (1.03–1.40)	Age, gender, race, ACS type, DM, smoking, TG, blood pressure, BMI, eGFR, treatment group, BNP, and CRP
Beatty 2012 [14]	USA	P	Stable CAD; 981 (81.5)	66.7 ± 11.0	MI, heart failure, or death	Quartile 4 vs. 1; > 35.6 vs. < 12.6 μg/ml	7.1	Total death:375; 1.77 (1.12–2.67); MACE:440; 1.43 (0.98–2.09)	Age, gender, race, DM, eGFR, beta-blocker, aspirin, statin, BMI, hemogloblin A1c, insulin, glucose, non-HDL-C, HDL-C, TG, LVEF, diastolic dysfunction, inducible ischemia, CRP, and NT-proBNP
Li 2012 [11]	China	P	CAD; 449 (68)	65.5 ± 10.9	Death, TVR, ACS, HF, and TIA/stroke	≥5.0 vs. < 5.0 μg/ml	1.6	MACE:109; 0.41 (0.23–0.73) ^a^	Age, gender, type 2 DM, hypertension, dyslipidemia, smoke, weight index, hsCRP, LVEF, creatinine clearance, TC, TG, HDL-C, LDL-C, fasting glucose, and coronary score
Lindberg 2012 [19]	Denmark	P	STEMI;735 (73.9)	63.0 ± 12.5	–	Quartile 4 vs. 1–3	2.3	Total death:99; 2.70 (1.30–5.6); CV death:50 2.57 (1.46–4.50)	Age, gender, smoking, hypercholesterolemia, DM, hypertension, BMI, CRP, peak troponin I, eGFR, multivessel disease, LVEF, and left anterior descending lesion (for CV death)
Delhaye 2013 [20]	France	P	Stable angina, NSTE-ACS; 477 (83.6)	62.5 ± 11	Death, MI or stroke	Tertile 3 vs. 1–2; > 20.1 vs. ≤ 20.1 μg/ml	3.7	MACE:82; 2.16 (1.35–3.46)	Age, DM, BMI, prior CAD, LVEF, creatinine, LDL-C, HDL-C, BNP, hs-CRP, and multivessel disease
Hascoet 2013 [21]	France	P	Stable CAD; 715 (100)	60.2 ± 8.0	–	≥7.3 vs. < 7.3 μg/ml	8.1	Total death:148; 1.71 (1.16–2.52); CV death:95 1.86 (1.11–3.13)	Age, smoking, waist, treatment for DM, GGT, apolipoprotein A1, resting heart rate, hsCRP, eGFR or history of kidney failure, BMI, fasting glucose, ankle–arm index, and case–control design
Pratesi 2016 [22]	Italy	P	Stable CAD; 138 (82)	59.4 ± 8.1	–	> 13.2 vs. ≤13.2 ng/ml	3.8	Total death:26; 6.5 (2.40–17.7)	Age, gender, previous PTCA, AF, PAD, NYHA class, Index of Disease Severity score, LVEF, eGFR, hemoglobin

*Abbreviations*: *HR* Hazard ratio, *RR* Risk ratio, *CI* Confidence intervals, *P* Prospective, *CV* Cardiovascular, *MI* Myocardial infarction; *STEMI* ST-segment elevation myocardial infarction, *ACS* Acute coronary syndrome, *IS* Ischemic stroke, *CI* Cerebral infarction, *TIA* Transient ischemic attack, *LVEF* Left ventricular ejection fraction, *HF* Heart failure, *PAD* Peripheral vascular disease, *CAD* Coronary artery disease, *HF* Heart failure, *CHF* Chronic heart failure, *PCI* Percutaneous coronary intervention, *CABG* Coronary artery bypass grafting, *TVR* Targeted vascular revascularization, *NYHA* New York Heart Association, *AF* Atrial fibrillation, *BNP* B-type natriuretic peptide, *NT-pro-BNP* N-terminal pro-B-type natriuretic peptide, *BMI* Body mass index, *eGFR* Estimated glomerular filtration rate, *LDL-C* Low density lipoprotein cholesterol, *HDL-C* High-density lipoprotein cholesterol, *TG* Triglycerides, *DM* Diabetes mellitus, *hsCRP* High sensitive C-reactive protein

^a^Results are calculated from the lowest versus the highest adiponectin level

**Table 2:** Quality assessment of the included studies

Author/Year	Representativeness of the exposed cohort	Selection of the non exposed cohort	Ascertainment of exposure	Demonstration that outcome was not present at study start	Comparability of cohorts on the basis of the design or analysis	Assessment of outcome	Enough follow-up periods (≥ 5 years)	Adequacy of follow-up of cohorts	TotalNOS
Pilz 2006 [7]	★	★	★	★	★	★	★	★	8
Shioji 2007 [8]	★	★	★	★	★★	★		★	8
Piestrzeniewicz 2008 [9]		★	★	★	★	★		★	6
Maiolino 2008 [12]	★	★	★	★	★	★	★	★	8
Huang 2010 [10]		★	★	★	★	★		★	6
Wilson 2011 [13]		★	★	★	★★	★		★	7
Beatty 2012 [14]		★	★	★	★★	★	★	★	8
Li 2012 [11]	★	★	★	★	★	★		★	7
Lindberg 2012 [19]		★	★	★	★★	★		★	7
Delhaye 2013 [20]	★	★	★	★	★	★		★	7
Hascoet 2013 [21]		★	★	★	★	★	★	★	7
Pratesi 2016 [22]		★	★	★	★	★		★	6

*NOS* Newcastle-Ottawa Scale

### Impact of elevated adiponectin level on MACE

**Fig. 2:**
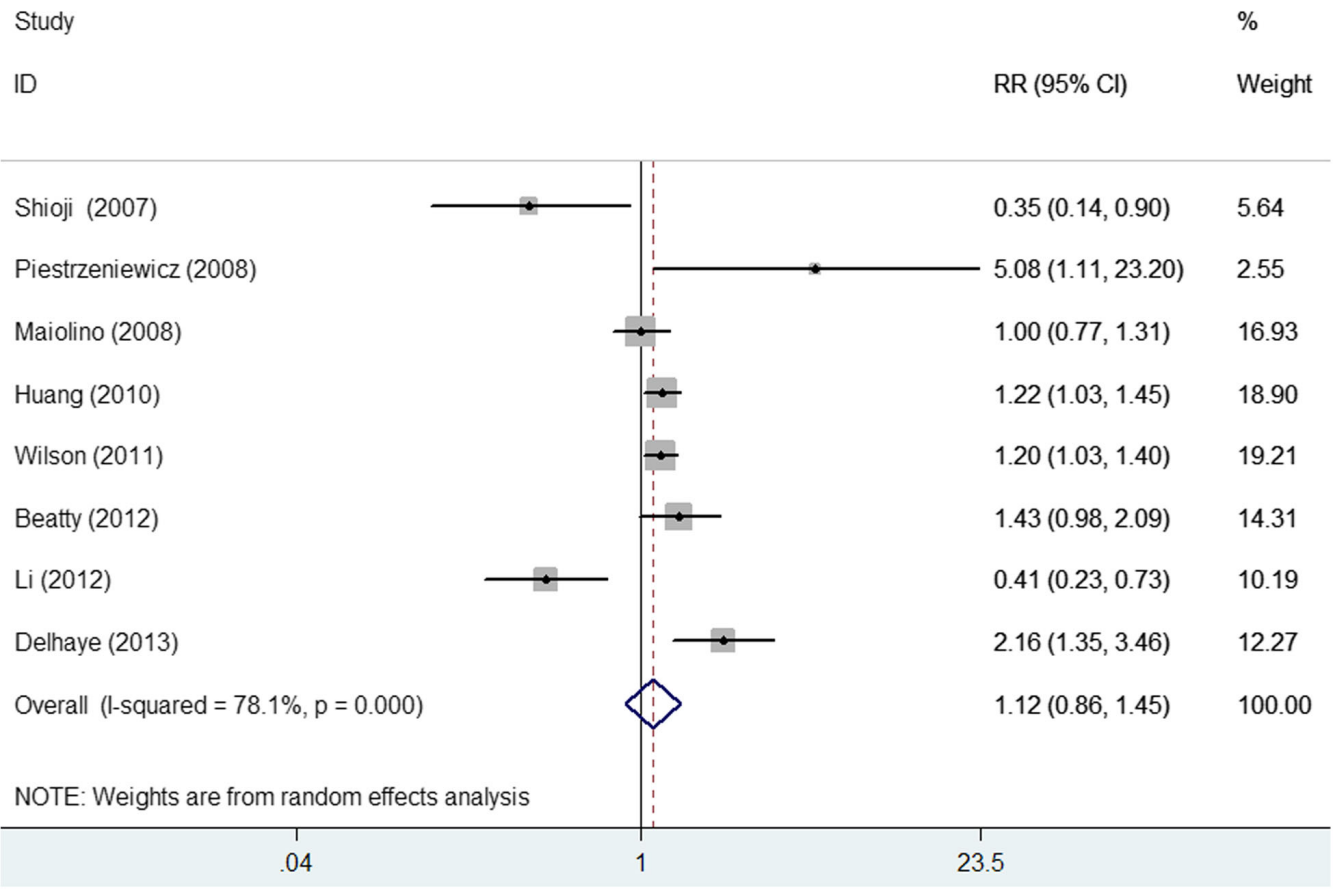
Forest plots showing pooled RR with 95% CI of major cardiovascular events for the highest vs. the lowest adiponectin level in a random effect model

Eight studies [8–14, 20] reported the association between elevated adiponectin level and MACE (Fig. 2). The pooled RR of MACE was 1.12 (95% CI 0.86–1.45; *p* = 0.407) for the highest vs the lowest category of adiponectin level in a random effect model, with significant heterogeneity (I^2^ = 78.1%; *p* < 0.001). Sensitivity analyses indicated no significant alterations in the original pooled risk estimate when any study was excluded from the analysis (data not shown). The Begg’s test (*p* = 0.902) and the Egger’s test (*p* = 0.746) did not show evidence of publication bias. Subgroup analysis showed that the pooled RR of MACE was 1.23 (95% CI 1.03–1.47; *p* = 0.020) and 1.31 (95% CI 1.02–1.68; *p* = 0.033) in patients with acute coronary syndrome (ACS) and follow-up more than 3 years, respectively. The detailed results of subgroup analysis are shown in Table 3.

**Table 3:** Subgroup analyses on major cardiovascular events

Subgroup	No. of studies	Pooled risk ratios	95% confidence intervals	Heterogeneity between studies
Sample sizes				
< 500	6	1.07	0.76–1.54	*p* < 0.001; I^2^ = 83.0%
≥ 500	2	1.16	0.82–1.65	*p* = 0.130; I^2^ = 56.5%
Type of patients				
All CAD	2	0.67	0.28–1.59	*p* = 0.006; I^2^ = 86.8%
ACS	3	1.23	1.03–1.47	*p* = 0.180; I^2^ = 41.7%
Follow-up duration				
< 3 years	4	0.84	0.36–1.97	*p* < 0.001; I^2^ = 86.5%
≥ 3 years	4	1.31	1.02–1.68	*p* = 0.038; I^2^ = 64.5%
Adjustment of LVEF				
Yes	5	1.24	0.72–2.12	*p* < 0.001; I^2^ = 84.1%
No	3	1.11	0.86–1.44	*p* = 0.034; I^2^ = 70.3%
Newcastle–Ottawa Scale				
≥ 7 stars	6	1.01	0.71–1.44	*p* < 0.001; I^2^ = 82.2%
< 7 stars	2	2.02	0.53–7.70	*p* = 0.068; I^2^ = 70.1%

*Abbreviations*: *ACS* Acute coronary syndrome, *CAD* Coronary artery disease, *LVEF* Left ventricular ejection fraction

### Impact of elevated adiponectin level on all-cause and cardiovascular mortality

**Fig. 3:**
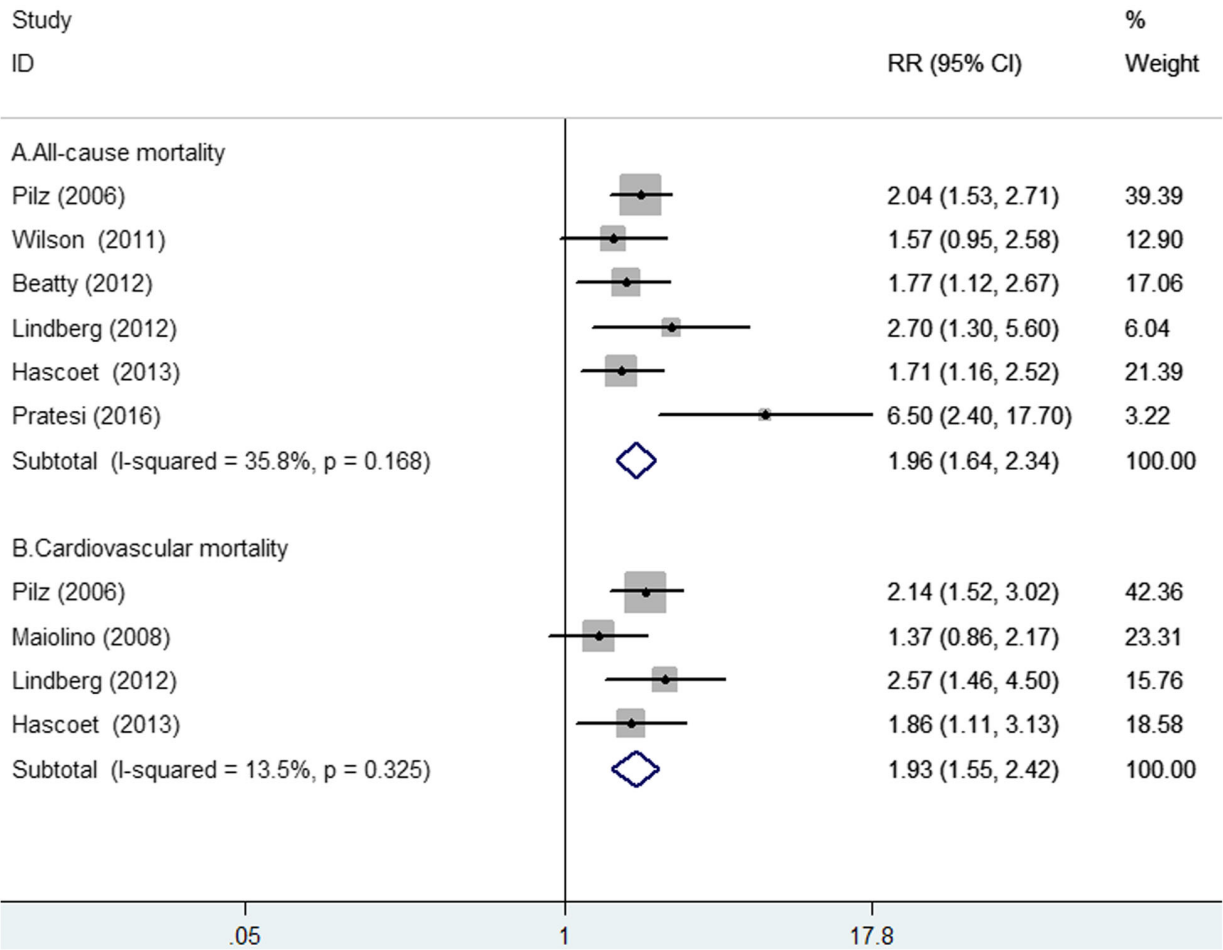
Forest plots showing pooled RR with 95% CI of all-cause (**a**) and cardiovascular mortality (**b**) for the highest vs. the lowest adiponectin level in a fixed-effect model

Elevated adiponectin level for predicting all-cause mortality was reported in 6 studies [7, 13, 14, 19, 21, 22] (Fig. 3a). The pooled RR of all-cause mortality was 1.96 (95% CI 1.64–2.34; *p* < 0.001) for the highest vs the lowest category of adiponectin level in a fixed-effect model, without significant heterogeneity (I^2^ = 35.8%; *p* = 0.168). Publication bias was not found according to the results of the Begg’s test (*p* = 0.348) and the Egger’s test (*p* = 0.194). Four studies [7, 12, 19, 21] reported the prognostic value of elevated adiponectin level for predicting cardiovascular mortality. As shown in Fig. 3b, a fixed-effect model was selected because there was no significant heterogeneity (I^2^ = 13.5%; *p* = 0.325).The pooled RR of cardiovascular mortality was 1.93 (95% CI 1.55–2.42; *p* < 0.001) for the highest vs the lowest category of adiponectin level. Sensitivity analysis slightly changed the original pooled risk estimates of all-cause or cardiovascular mortality (data not shown).

## Discussion

The current meta-analysis suggests that an elevated total adiponectin level is possibly an independent predictor of cardiovascular and all-cause mortality in CAD patients. When compared with those with the lowest circulating total adiponectin level, CAD patients with the highest total adiponectin level exhibited approximately 93% and 96% higher risk of cardiovascular and all-cause mortality, respectively. These findings highlight the importance of detecting adiponectin level for the prediction of cardiovascular and all-cause mortality in CAD patients. However, elevated total adiponectin level was not associated with an increased risk of MACE in CAD patients.

The association of adiponectin level with cardiovascular and all-cause mortality risk has been confirmed in previous meta-analysis [5, 6]. However, these two meta-analyses did not focus on the CAD patients. Similarly, our study demonstrated the prognostic role of adiponectin for predicting cardiovascular and all-cause mortality in CAD patients. Results from the previous and our meta-analyses raise a clinical question that whether reducing adiponectin level by medication may improve the survival outcomes of CAD patients. Moreover, we further investigated the association of elevated adiponectin level with MACE risk. Overall, our meta-analysis did not observe a significant association between elevated adiponectin level and MACE.

CAD is a heterogeneous clinical conditions, which spans from angina pectoris to acute myocardial infarction. Adipokine level varies acoss the type of CAD. Serum adiponectin level was lower in AMI patients as compared to stable CAD patients [23]. In subgroup analyses, there was a close association of elevated adiponectin level with MACE risk in this subgroup with ACS patients. A potential explanation for this finding may be that higher level of adiponectin in ACS represents a compensatory response to against acute inflammatory and hypoxia-reoxygenation lesions [24]. Moreover, the effect of elevated adiponectin follow-up duration level on MACE was strengthened with the lengthening of the follow-up duration.

Several studies analyzing blood adiponectin level by continuous data also showed a significant association of elevated adiponectin level with adverse cardiovascular outcomes. A prospective study [25] indicated that per 1 standard deviation increment of log-transformed plasma adiponectin was independently predictive of the subsequent risk of all-cause mortality, cardiac mortality, and myocardial infarction in CAD patients. In patients with ACS, adiponectin was associated with higher risk for MACE (adjusted HR 1.08/increment of 1000) [26]. In addition, per tertile increase [27] or unit increment of log-transformed [28] adiponectin level also predicted all-cause mortality in patients with AMI. These findings further supported the prognostic role of adiponectin level for predicting adverse outcomes in CAD patients.

The mechanisms underlying the prognostic utility of adiponectin in CAD patients remain unclear. One potential explanation is that elevated adiponectin level may reflect the severity of coronary lesions. Another explanation for the prognostic role of elevated adiponectin level with worse clinical outcomes may be adiponectin resistance [29, 30].

Several potential limitations of this meta-analysis should be noted. First, measurement of adiponectin level at baseline and lack of serial determination may have led to misclassification of the category of patients. Second, the cutoff value of adiponectin elevation varied between studies and we failed to determine the optimal threshold of higher adiponectin level. Third, significant heterogeneity was observed in pooling MACE outcome. Different subtypes of CAD, threshold of adiponectin elevation and length of follow-up were partly responsible for between-study heterogeneity. Fourth, due to the distinct units of adiponectin level increment, we did not evaluate the prognostic value of adiponectin level by continuous data. Finally, our meta-analysis only investigated the prognostic value of total adiponectin level and more studies are necessary to examine the prognostic role of various isoforms of adipocytokines, such as leptin, visfatin and resistin or the high–molecular weight isoform [31].

## Conclusions

This meta-analysis demonstrates that elevated total adiponectin level is possibly an independent predictor of cardiovascular and all-cause mortality in CAD patients. Measurement of total adiponectin level has potential to improve the prediction of cardiovascular and all-cause mortality in these patients. Future studies are warranted to evaluate the prognostic significance of adiponectin level is different between ACS and stable CAD patients.

## Supplementary information

**Additional file 1.** Search strategy developed for the meta-analysis.

## Data Availability

All data generated or analyzed during this study are included in this article.

## Abbreviations

ACS: Acute coronary syndrome
CAD: Coronary artery disease
CI: Confidence interval
MACE: Major adverse cardiovascular events
NOS: Newcastle-Ottawa Scale
RR: Risk ratio
