# Supplementary material for: Prognostic value of adiponectin level in patients with coronary artery disease: a systematic review and meta-analysis
Source: Lipids Health Dis. 2019 Dec 23;18:227. doi: 10.1186/s12944-019-1168-3 (PMC6929433; doi:10.1186/s12944-019-1168-3)
Supplement: Supplementary file 1 — Additional file 1. Search strategy developed for the meta-analysis. [file 12944_2019_1168_MOESM1_ESM.doc]

**Additional file 1 – Search strategy developed for the meta-analysis**

**Medical Databases---Pubmed** (Publications until May 10, 2019)

# 1 (“adiponectin” [Free terms])

# 2 (“coronary artery disease” [MeSH Terms] OR “coronary heart disease” [MeSH Terms] OR myocardial infarction [MeSH Terms] OR “acute coronary syndromes” [MeSH Terms] OR “angina” [MeSH Terms])

# 3 (“all-cause mortality” [MeSH Terms] OR cardiovascular mortality [MeSH Terms] OR “death” [Free terms] OR “mortality” [Free terms] OR “cardiovascular events” [Free terms])

# 4 (#1) OR (#2) OR (#3).

**Medical Databases--- Embase** (Publications until May 10, 2019)

# 1 (“adiponectin” [All Fields])

# 2 (“coronary artery disease” [All Fields] OR “coronary heart disease” [All Fields] OR myocardial infarction [All Fields] OR “acute coronary syndromes” [All Fields] OR “angina” [All Fields])

# 3 ( “death” [All Fields] OR “mortality” [All Fields] OR “cardiovascular events” [All Fields])

# 4 (#1) OR (#2) OR (#3).
